# Case report of concomitant avulsion fractures of the medial meniscus and posterior cruciate ligament

**DOI:** 10.1097/MD.0000000000028273

**Published:** 2021-12-17

**Authors:** Bertan Cengiz, Sinan Karaoglu

**Affiliations:** Acibadem Kayseri Hospital Orthopaedics and Traumatology Clinic, Kayseri, Turkey.

**Keywords:** concomitant, medial meniscus avulsion fracture, PCL avulsion fracture, posterior approach

## Abstract

**Rationale:**

Posterior cruciate ligament (PCL) is the strongest ligament of the knee, and avulsion fractures of PCL are a very rare type of injury. These injuries occur as a result of high-energy traumas, and different accompanying pathologies may be seen. However, tibial avulsion fracture of the PCL associated with a medial meniscus (MM) avulsion fracture has never been reported before. We want to present this unique type of posteromedial knee injury as a case report.

**Patient concern:**

A 42-year-old man presented with severe pain and swelling due to a ski injury.

**Diagnosis:**

Concomitant avulsion fractures of PCL and MM were detected after imaging.

**Interventions:**

Both avulsion fractures were treated with open reduction and fixation with lag screws using the posterior approach.

**Outcomes:**

No complications were encountered, and the painless full range of motion and weight-bearing was achieved at the third month after the operation.

**Lessons:**

Anatomical reduction and stable fixation of these intra-articular fractures are essential for the stability of the knee. The posterior approach should be kept in mind to access these types of fractures safely. Care should be taken in terms of other injuries that may accompany the PCL avulsion fractures caused by high-energy traumas.

## 1 Introduction

The menisci are located between the opposite surfaces of the femur and the tibia to reduce axial loads on the joint and shock absorption. Bony attachments of the menisci prevent the extrusion of the meniscus.^[1]^ Bony or soft tissue root avulsion injuries or radial tears within 1 cm of meniscus root attachment are described as meniscal root tears. According to the meniscus root tears classification of LaPrade, the posterior meniscus root's bony avulsion is defined as Type V.^[2]^

Meniscus root avulsion fracture is a very rare pattern of meniscus root injuries. All cases of meniscal root avulsion fractures previously reported in the literature are developed as a result of severe trauma.^[3]^

While posterior cruciate ligament (PCL) injuries are rare injuries, PCL avulsion fractures are an extremely rare type of this injury.^[4]^ The injury mechanism is usually due to a direct force displacing the proximal tibia posteriorly while the knee is flexed. Another common type of injury mechanism is hyperextension of the knee.^[5]^

Anterior cruciate ligament (ACL) ruptures concomitant lateral meniscal root tears are a common type of injury.^[6]^ Depending on their anatomical relationship, PCL ruptures may be associated with meniscus lesions, especially with medial meniscus posterior horn root tears.^[7]^

According to our current knowledge, a concomitant avulsion fracture of the medial meniscus and PCL has not been described as well. We report a 42-year-old man with a tibial avulsion fracture of the PCL accompanying medial meniscus avulsion fracture in this case study.

## 2 Case presentation

A 42-year-old man was admitted to emergency service after a ski injury. He was unable to weight-bear. Effusion of the knee, severe posterior knee pain, and tenderness of the posterior structures of the knee were determined in the initial physical examination. The pain was getting worse when the knee was extended. The range of motion (ROM) was very limited, and the motion itself was causing the patient a lot of pain. The patient did not allow the tests Lachmann, McMurray, or posterior drawer to be performed due to pain and spasm. There were no findings of neurovascular damage.

**Figure 1:**
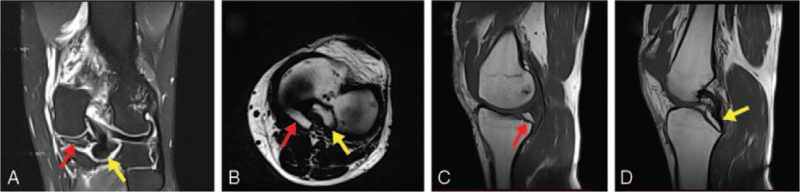
Preoperative magnetic resonance images findings of the knee (A) Coronal T2 FS image, the red arrow shows avulsion fracture of the medial meniscus, and yellow arrow shows avulsion fracture of the posterior cruciate ligament (PCL). (B) Axial T1 image, the red arrow shows avulsion fracture of the medial meniscus, and yellow arrow shows avulsion fracture of the PCL. (C) Sagittal PD image, the red arrow shows avulsion fracture of the medial meniscus. (D) Sagittal PD image yellow arrow shows avulsion fracture of the PCL.

Two avulsed fragments were revealed in the plain radiographs (Fig. 3A&B). MRI showed the fragments belonged to PCL and lateral meniscus, and concomitant avulsion fractures of the medial meniscus and PCL were diagnosed (Fig. 1). ACL, lateral meniscus and medial/lateral collateral ligaments were intact. Based on these radiological and clinical findings, and due to the instability and locking risk that simultaneous PCL and medial meniscus avulsion fracture would create in the knee, the patient was recommended to operate.

The operation was performed the day after the injury. Under general anesthesia, physical examination was performed again, and the posterior drawer test was positive with a soft endpoint. Lachmann test was negative, and valgus stress test when the knee flexed 30° was positive as grade I laxity (opening of the medial joint <5 mm). Varus/valgus stress radiographs were performed under fluoroscopy, and medial space opening was checked and confirmed.

**Figure 2:**
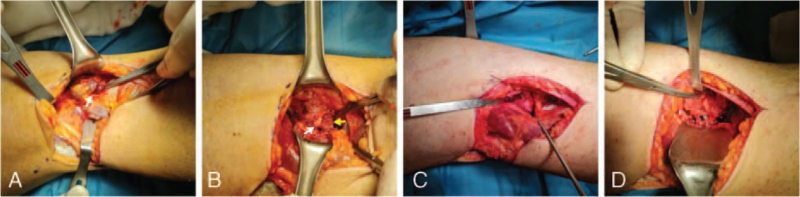
Intraoperative images (A) The varicose vessel which enlarged over the capsule. (B) Avulsed fragments of the fractures of posterior cruciate ligament (PCL) and medial meniscus, White arrow shows PCL avulsion fracture, and yellow arrow shows medial meniscus avulsion fracture. (C) Fixation of avulsion fracture of the medial meniscus with compression screw. (D) Fixation of the avulsion fracture of PCL with cancellous screw with washer.

Then the patient was placed in the prone position, and a tourniquet was applied and inflated. An L-shaped incision was made over the fossa poplitea. Dissection was performed, and the interval between the semimembranosus and medial head of the gastrocnemius was used, as described by Burks and Schaffer.^[8]^ At that point, a varicose vein that enlarged and curved, that we thought it was caused by trauma, was detected over the capsule; a cardiovascular surgeon was attended to the surgery and ligated the veins (Fig. 2A). And then gastrocnemius muscle was retracted laterally, and the posterior capsule was exposed. The avulsed bone fragments were palpated over the capsule, and a vertical capsular incision between these fragments was made (Fig. 2B). After removing the bone clots and soft tissues around the avulsed fragments, bone beds were prepared. Firstly, the medial meniscus's avulsion fracture was reducted and fixed with two Kirschner wires temporarily, and then PCL avulsion fracture was reducted in the same way under fluoroscopy control. Later, permanent fixation of the avulsion fracture of the medial meniscus was done with two headless 3.0 mm compression screws after drilling and measurement (Fig. 2C). Then, the PCL avulsion fracture was fixed with a 4.0 mm partially threaded, cannulated, cancellous screw with a washer (Fig. 2D). The fixation of the fragments was confirmed under fluoroscopy again, and the posterior drawer test was performed gently to check stability. After the irrigation of the wound, the capsule, subcutaneous layers, and skin was closed. A hinged, long leg brace was applied and locked in 0° knee extension.

**Figure 3:**
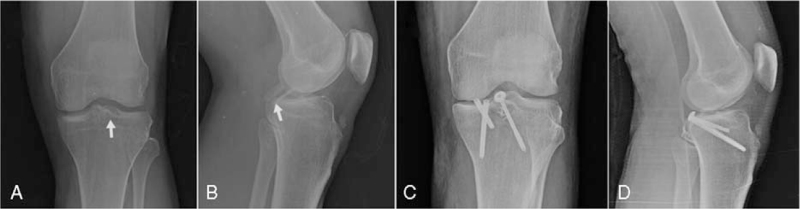
X-rays of the knee. (A) Preoperative anteroposterior X-ray, white arrow shows avulsion fracture of the posterior cruciate ligament. (B) Preoperative lateral X-ray, white arrow shows avulsion fracture of the medial meniscus. (C) Postoperative AP X-ray. (D) Postoperative lateral X-ray.

The knee was kept in full extension with the hinged brace for two weeks and instructed for leg raises only in the brace several times daily. On the third week, isometric and passive ROM exercises with continuous passive motion device were applied and gradually increased as tolerated. Weight-bearing was not allowed for six weeks. At postoperative controls, knee X-rays were taken, and fracture healing was followed (Fig. 3C&D). No complications were encountered in the follow-up of the patient. Full weight-bearing and ROM without pain were achieved in the third month after the operation. The visual analog scale score was 8 before surgery and 2 in the third month after the surgery. Lysholm score was 14 before surgery and 85 in the sixth month after the surgery.

## 3 Discussion

PCL is the strongest ligamentous structure of the knee and is known to be twice as strong as ACL.^[9]^ Therefore, PCL takes on one of the most essential roles in knee stabilization, and treating PCL injuries is of paramount importance. The most common cause of PCL injuries is motorcycle accidents, and it is caused by force pushing the proximal tibia directly to the posterior while the knee is flexed. It is more common in Eastern countries such as China and India, where two-wheeled vehicles are frequently used.^[10]^ Also, sports injuries can cause PCL ruptures due to knee hyperextension. In the systematic review of Hooper et al including 637 patients with tibial side PCL bony avulsion, 68.4% of the patients have sustained the injury from a motorcycle accident, and 16.6% from sports-related injury.^[5]^ According to the literature, femoral-sided avulsions are more common in pediatric patients; however, tibial-sided bony avulsions are the most common type of PCL avulsion fractures in general.^[4]^

Because PCL avulsion fractures are high-energy traumas, they can often be accompanied by different pathologies. 16.8% concomitant meniscal injuries and 19.1% concomitant ligamentous injuries were reported in the study of Hooper et al., and 57.5% of the meniscal injuries were medial.^[5]^ Various ligamentous pathologies accompanying PCL injuries have been reported with a rate of up to 96.5%.^[11]^ Up to date, there was no tibial PCL avulsion fracture concomitant medial meniscus root avulsion fracture reported yet. Our patient experienced this injury due to a fall accident while skiing. Although we couldn’t get explicit information on the injury mechanism, we think it was exposed to rotational forces when the knee is flexed, with force to the proximal tibia displacing posteriorly.

Meniscal root avulsion fractures are also rare injuries; therefore, only a small number of literature is available as case reports. Feucht stated 12 meniscal root avulsions had been reported up to 2013 and 92% of these injuries are medial meniscus posterior root avulsion. Only two patients (17%) concomitant injuries were found, and these are one ACL avulsion fracture and one ACL tear.^[3]^

It was hypothesized that in younger patients, the meniscal root ligament has greater tensile strength than the adjacent bone, so that an avulsion fracture is more likely than root tear.^[12]^ Reported case reports in the literature support this hypothesis.^[12–15]^ For skeletally mature patients, there is a different hypothesis about the injury mechanism of the meniscal root still under investigation. Anterior displacement force applied to the proximal tibia with external rotation produced high forces at the posterior medial root due to the pressure of the posteromedial femoral condyle to the posterior horn of the medial meniscus. In the absence of the ACL, this causes further pressure to increase. This mechanism described by Markolf et al could explain the posteromedial meniscal root avulsion in the adult patient group. However, our patient's injury mechanism is different from those described so far, as it was the first case of simultaneous avulsion fracture of PCL and the medial meniscus.

PCL injuries can be treated conservatively as well, and good results could be achieved as reported. Mid-term and long-term (>10 years) results were reported in isolated injuries of PCL, and clinical and functional outcomes were reported as good.^[16,17]^ In these studies, the injury pattern was not the avulsion fractures of PCL, and no concomitant lesions were described. However, conservatively treated PCL avulsion fracture outcomes were not acceptable as the studies above.^[18]^

PCL insufficiency alone leads to severe instability; also, medial meniscus avulsion was accompanied in our case. To restore stability and prevent mechanical symptoms such as locking, we thought surgical fixation is the proper treatment method for this case. Open and arthroscopic fixation techniques were described for the fixation method of both PCL and meniscus avulsion fractures.^[13,19–21]^ We decided to perform both PCL and meniscus fixation with the posterior approach described by Burks et al.^[8]^ In this approach, the interval between the semimembranosus and medial head of the gastrocnemius is used, and neurovascular structures were protected by retracting the gastrocnemius laterally. The visualization and management of the fracture are more challenging than other previously described approaches, but allowing faster rehabilitation protocol is advantageous.^[22]^ A new minimally invasive approach targeting the fracture zone, using the interval between two heads of the gastrocnemius, was recently described by Gavaskar et al and no complications were reported in their study, including 22 patients with PCL avulsions. Minimizing surgical dissection and recovery time and faster rehabilitation process were reported as the advantages of this procedure.

Despite the good results of the open technique, the arthroscopic approach has become more popular in recent years. It is more advantageous to diagnose and treat the concomitant intraarticular pathology, decrease the risk of wound problems, and allow early rehabilitation. Different portals, fixation materials, and techniques are described about the arthroscopic procedure.^[5]^ We preferred the open posterior approach to manage the PCL and meniscus avulsion fractures better simultaneously.

The most comprehensive review about the comparison of the open and arthroscopic techniques of PCL avulsion fractures was reported by Hooper et al.^[5]^ They stated that both techniques’ success and complication rates are similar to each other. The arthroscopy group had higher functional scores than the open group; however, arthrofibrosis was slightly more common in the arthroscopic group. Another study reported by Sabat et al compared the outcomes of PCL avulsion injuries, and it was stated that the open reduction and screw fixation group had similar good outcome scores as the arthroscopic approach and suture fixation group, but less residual laxity was seen in the arthroscopy group.^[23]^ The main complication stated for both open and arthroscopic surgery is arthrofibrosis. In the arthroscopy group, one case at least that reported arthrofibrosis as a complication ranges from 6.3% to 35.7%.^[5]^ Lamoria et al reported three arthrofibrosis of 22 patients in the study of arthroscopic suture fixation method for PCL avulsion fractures from the tibial side, and all were treated with manipulation under general anesthesia and did not require surgical debridement.^[24]^ In the open approach group, a total of 8 cases were reported as arthrofibrosis in 20 studies.^[5]^ Prolonged time-to-surgery is reported as a leading factor for arthrofibrosis.^[25]^

In conclusion, PCL avulsion fractures are rarely seen due to severe trauma, and determining other concomitant injuries of the knee is essential. Simultaneous avulsion fractures of PCL and medial meniscus were firstly reported in the literature by this case presentation. Recovery rates of these pathologies after successful surgical intervention are very high. We believe that rapid surgical intervention after diagnosis increases treatment success in such complex injuries. The open posterior approach that we used for this case is a safe and reproducible method for this kind of injury.

## Author contributions

**Conceptualization:** Bertan Cengiz.

**Data curation:** Bertan Cengiz.

**Formal analysis:** Bertan Cengiz.

**Methodology:** Bertan Cengiz.

**Supervision:** Sinan Karaoglu.

**Writing – original draft:** Bertan Cengiz.

**Writing – review & editing:** Sinan Karaoglu.
